# The Spatial Distribution Characteristics of Soil Organic Carbon and Its Effects on Topsoil under Different Karst Landforms

**DOI:** 10.3390/ijerph17082889

**Published:** 2020-04-22

**Authors:** Xingfu Wang, Xianfei Huang, Jiwei Hu, Zhenming Zhang

**Affiliations:** 1 School of Karst Science, Guizhou Normal University, Guiyang 550001, Guizhou, China; wang88xingfu@163.com (X.W.); jiweihu@yahoo.com (J.H.); 2 Guizhou Provincial Key Laboratory of Information System of Mountainous Areas and Protection of Ecological Environment, Guizhou Normal University, Guiyang 550001, Guizhou, China; 3 Institute of Biology, Guizhou Academy of Sciences, Guiyang 550001, Guizhou, China; zhang6653579@163.com

**Keywords:** soil organic carbon (SOC), spatial heterogeneity, impact factor, effect mechanism, different karst landforms, Guizhou Province in SW China

## Abstract

Karst landforms are widely distributed in Guizhou Province, and the karst terrain is complex. To investigate the spatial distribution characteristics of soil organic carbon (SOC) in topsoil in different karst landforms, a total of 920 samples were taken from different karst landforms. The study areas, Puding, Xingyi, Guanling, Libo and Yinjiang in Guizhou Province, represent the karst plateau (KP), karst peak-cluster depression (KPCD), karst canyon (KC), karst virgin forest (KVF) and karst trough valley (KTV) landforms, respectively. The characteristics of the SOC contents in areas with different vegetation, land use and soil types under different karst landforms were analyzed. The dimensionality of the factors was reduced via principal component analysis, the relationships among SOC content and different factors were subjected to redundancy analysis, and the effects of the main impact factors on SOC were discussed. The results showed that there was a large discrepancy in the SOC contents in the topsoil layers among different types of karst landforms, the changes in the SOC content in the topsoil layer were highly variable, and the discrepancy in the upper soil layer was higher than that in the lower soil layer. The SOC contents in the 0–50 cm topsoil layers in different karst landforms were between 7.76 and 38.29 g·kg^−1^, the SOC content gradually decreased with increasing soil depth, and the descending order of the SOC contents in different karst landforms was KTV > KVF > KC > KPCD > KP.

## 1. Introduction

Soil is ecologically complex as it is affected by climate, biology, topography and other factors; moreover, soil connects the hydrosphere, lithosphere, biosphere and atmosphere [1]. Soil organic carbon (SOC) is an important basis for soil fertility, and it plays a key role in improving soil quality and promoting increased agricultural output [2]. According to the statistics on the global organic carbon stock, the reserve of SOC in the soil horizon (0–100 cm) globally reaches 1460 Pg [3]. The reserves of the SOC reservoir are three times greater than those of the vegetation organic carbon reservoir and two times greater than those of the atmospheric carbon reservoir [4,5], which indicates that SOC plays an important role in the global carbon cycle. Because there are large reserves of organic carbon in soil reservoirs, small changes in SOC may lead to changes in the global climate [6]. Because SOC is an important part of the terrestrial carbon pool, it is the main factor affecting the carbon balance in terrestrial carbon cycle systems [7]. Therefore, the conversion of SOC has a direct impact on the stores of carbon on land, plant growth and soil fertility, and it is an important indicator of the global carbon cycle [8]. The main composition of rock in karst areas is carbonate rocks, and carbonate is the largest reservoir of carbon storage in the world [9]. The problem of rocky desertification in karst area is serious. In the process of karst rocky desertification, the change in SOC content has a significant impact on the global carbon cycle. Therefore, research on SOC in karst areas has become one of the hot topics in the world [10,11].

Karst areas are unique ecosystems that differ from non-karst areas in terms of terrain and landforms because of the special geological and climatic conditions that lead to karst mountainous areas exhibiting low environmental capacity, low stability and poor self-regulation [12,13]. In different karst regions of Guizhou, the soil environment is coupled with a high rate of rock exposure and a noncontinuous soil layer, and the microtopography is complex, which leads to the development of noncontinuous shallow soil with varying thickness. These factors complicate the calculations of the SOC contents in different karst landforms. Due to the unique characteristics of ecological systems in different karst landform areas, the method used to estimate the SOC contents in non-karst regions is not suitable for these special areas [14]. Many scholars have studied the characteristics of environmental factors that influence the spatial distribution heterogeneity of SOC. Currently, some indexes, such as soil thickness, soil bulk density and rock exposure rate, have been considered to determine the spatial diversity of SOC in karst areas, while other factors have not been considered [15,16]. For example, the soil bulk density differs among different soil types, and the greater the difference in the soil bulk density is, the greater the difference in SOC content [17]. The distribution characteristics of SOC were found to be significantly related to terrain attributes and the normalized difference vegetation index, and the SOC spatial distribution pattern was found to be controlled by cultivation activity and topography [18]. Vegetation conversion leads to a significant change in the SOC content, and its succession enhances the accumulation of carbon in the soil [19,20]. In soil around mineral areas under forests, natural canopy disturbance may have positive effects on SOC [21]. In addition, the SOC content in karst mountainous areas is influenced by land use, agricultural succession and other factors [22,23]. There is high variance in the SOC content in the top several soil layers, and the SOC gradually decreases as the soil depth increases. Especially at depths of 0–50 cm, the SOC content significantly decreases, but in soil horizons of 50–100 cm, SOC is relatively stable [24,25]. The content of SOC is influenced by multiple environmental factors and its high spatial heterogeneity in karst mountainous areas, so sensitive methods to calculate SOC content are essential [26]. Obviously, many scholars have researched the diversity in the spatial distribution of the SOC content and its impact factors in karst mountainous areas [27,28,29]. However, there are few reports about the spatial heterogeneity of the SOC distribution and its impact factors in topsoil under different karst landforms.

The spatial heterogeneity of the SOC distribution is affected by multiple environmental factors, and these influencing processes are poorly understood. Therefore, the estimates of SOC contents by different people may be quite different. Therefore, the correlation between the spatial heterogeneity of SOC and different factors should be analyzed by investigating the spatial variability in related factors, such as soil bulk density, rock exposure, rock content, slope aspect, land use and vegetation types. It is important to revise the relationship between SOC and environmental factors to improve the reliability of estimates of the SOC content in different karst landforms.

## 2. Materials and Methods

### 2.1. Study Region

The study region (103°36′ E–109°35′ E, 24°37′ N–29°13′ N) is as follows: Puding County, Guanling County, Xingyi County, Yinjiang County and Libo County in Guizhou Province in SW China. The study region is located in the slope belt of the transition zone between the Yunnan–Guizhou Plateau and the Sichuan Basin and Hunan hills. The total area of Guizhou Province is 0.176 million km^2^, but the karst area accounts for 61.56% of the entire province, and rocky desertification accounts for 34.59%. Guizhou is in a subtropical region with a humid monsoon climate, and it is affected by the quasi-stationary Guiyang–Kunming front and the reduced cold air mass from Siberia moving to south China. The average annual temperature is between 13 and 16 °C, and the annual accumulated temperature is over 5500 °C. The elevation is between 147.7 m and 2900.6 m above sea level, its average value is approximately 1100 m, and the rainfall is between 1150 and 1250 mm. In accordance with the survey and statistics, three soil groups were identified in different karst landforms: limestone soil, paddy soil, and yellow soil. Vegetation includes walnut, *Cyclobalanopsis glauca* trees, pine trees, strawberry trees, plum trees and Chinese pear. The main crops and grasses are sweet potato, paddy rice, corn, pepper, soybean and foxtail grass.

### 2.2. Sampling Point Layout

**Figure 1:**
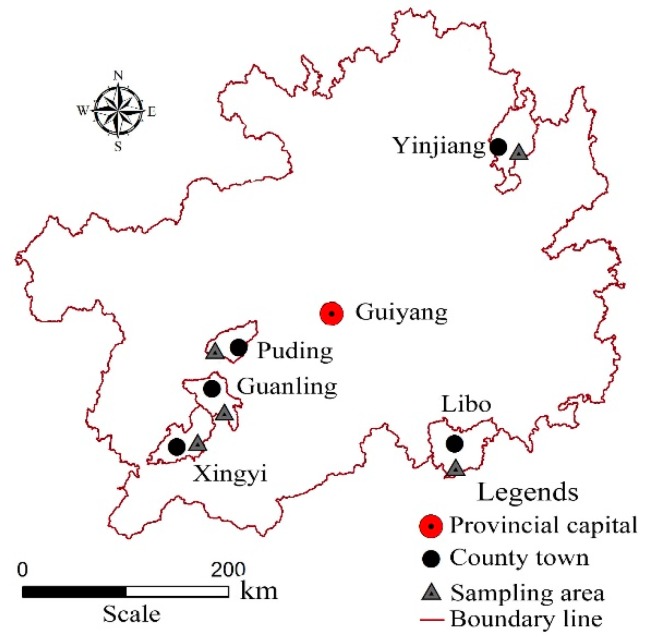
Distribution of sampling points.

To research the different SOC spatial distributions in different karst landforms, the study area included Maguan in Puding County, Wanfenglin in Xingyi County, Huajiang Canyon in Guanling County, virgin forest area in Libo County and the trough valley in Yinjiang County. The areas represent the karst plateau (KP), karst peak-cluster depression (KPCD), karst canyon (KC), karst virgin forest (KVF) and karst trough valley (KTV) (Figure 1). According to the distribution of karst landforms, a total of 152 sample spot profiles were sited in different karst landforms. At each site, the soil profile was divided into 7 layers (0–5, 5–10, 10–15, 15–20, 20–30, 30–40 and 40–50 cm).

### 2.3. Soil Sample Collection and Test

The actual soil thickness was less than 50 cm at many of the sampling sites, which resulted in the actual sample numbers being lower than the number of theoretical samples. A total of 920 samples were taken from different soil horizons in different karst landforms by systematic methods. Among these samples, 344 samples in 53 soil profiles were taken from the KP, 147 samples in 22 soil profiles were taken from the KPCD, 143 samples in 25 soil profiles were taken from the KC, 136 samples in 25 soil profiles were taken from the KVF and 150 samples in 27 soil profiles were taken from the KTV. The relative information about each sampling point, including the soil bulk density, rock exposure and other factors, was recorded and measured at the time of sampling. The soil bulk density (SBD) of each soil layer was determined via the cutting ring method [17]. The rock exposure rates around the sampling sites were assessed by the line-transect method, and the line length was set to 10 m [24,27]. Soil samples were packed into sealed bags and numbered, and the weight of each sample was approximately 1 kg. The samples were taken to the laboratory, and all soil samples were air-dried, ground, sieved to remove the gravel fraction (>2 mm), weighed and prepared as required for laboratory analysis. The total SOC content in the soil samples was determined using the potassium dichromate method: K_2_Cr_2_O_7_ oxidation at 170–180 °C followed by titration with FeSO_4_ [30].

### 2.4. Calculations and Statistical Analysis

#### 2.4.1. The Calculation of SBD

The SBD (g·cm^−3^) was tested at the time of sampling by the cylindrical core method. The formula is as follows:

(1) $$\mathrm{SBD}=\frac{M_{2}-M_{1}}{V}$$

where *M_1_* is the weight of the cutting ring (g), *M_2_* is the weight of the cutting ring with dry soil (g), *V* is the volume of cutting ring (cm^3^).

#### 2.4.2. Analysis Methods

Factor analysis was carried out via principal component analysis (PCA) and redundancy analysis (RDA). PCA analyzes the similarity and diversity among different factors and distinguishes the significant impact factors from all samples. RDA is an important method of constrained ordination, and it can sort the datasets of species and environmental factors and sort the environmental factors under constrained species. RDA analyzes environmental factors in response to the structure of species, not only reflecting the correlation among samples, species and environmental factors.

The aims of PCA and RDA are to find a new variable instead of an old variable; fundamentally, they are both dimension-reduction analyses, but they are also different. PCA, as an unsupervised dimension-reduction method, only needs to decompose the characteristic values and can numerically compress and remove noise. The PCA calculation method is simple and easy to implement, but there is some ambiguity in the of meaning of each feature dimension of PCA analysis, and the interpretability of PCA results is not as strong as that of the features in the original sample. The advantages of RDA are that the species response to environmental factors can be considered, and the species distribution is constrained by specific factors (e.g., altitude, slope and vegetation).

## 3. Results and Analysis

### 3.1. The Statistical Characteristics of Soil Properties and SOC in Different Karst Landforms

#### 3.1.1. The Spatial Distribution Characteristics of Soil Properties

**Figure 2:**
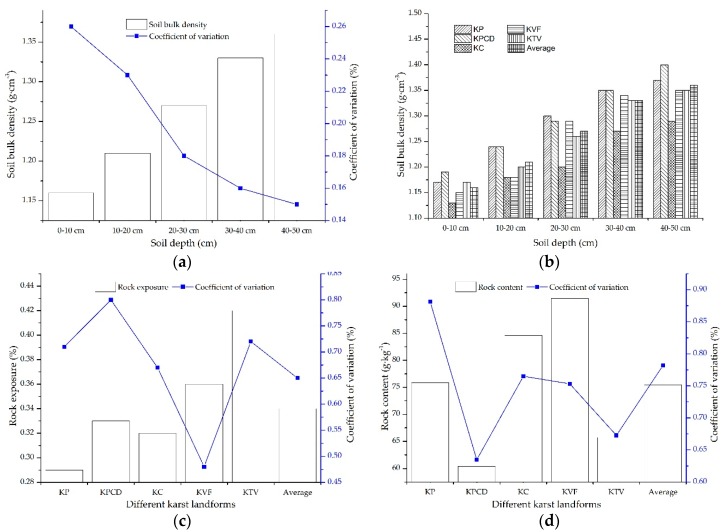
Information on soil bulk density (SBD), rock exposure and rock content: (**a**) shows the SBD in different soil layers; (**b**) shows the SBD in different karst landforms; (**c**) and (**d**) represent the rock exposure and rock content in different karst landforms, respectively.

The main soil properties in karst mountainous areas include SBD, rock exposure and rock content, which are three important factors that affect SOC in karst areas [18,24,27]. The spatial distribution characteristics of SBD, rock exposure and rock content are shown in Figure 2. The average SBD in the 0–10 cm soil layer is 1.16 g·cm^−3^, and the SBD increases with soil depth, reaching 1.40 g·cm^−3^ in the 40–50 cm soil layer. The coefficient of variation of SBD in each soil layer decreases with soil depth, and the changes in SBD in the 0–10 cm and 10–20 cm soil layers exhibit moderate variations, but in the soil layers below 20 cm, there is low variability [31], as shown in Figure 2a. The soil bulk densities in the KP and KPCD are generally higher than those in other karst landforms and the average values; the soil bulk densities in the KTV and KVF are similar to the average values, and the soil bulk densities in each KC soil layer are the lowest (Figure 2a). The rock exposure rates in the different karst landforms are 0.29, 0.33, 0.32, 0.36 and 0.42 in the KP, KPCD, KC, KVF and KTV, respectively. The rock exposure rates in the KTV and KVF are higher than the average rate of 0.34. In descending order, the rock exposure changes in the different karst landforms are KPCD > KTV > KP > KC average value > KVF, and the coefficient of variation of rock exposure is highly variable among the different karst landforms (Figure 2c). The rock contents in the different karst landforms are 75.82, 60.39, 84.63, 91.44 and 65.72 g·kg^−1^ in the KP, KPCD, KC, KVF and KTV, respectively, and the rock content changes in the different karst landforms in descending order are KP > average value > KC > KVF > KTV > KPCD. The rock contents in the KC, KVF and KP are higher than the average value, and the coefficient of variation of the rock content is highly variable (Figure 2d). In summary, there are high discrepancies in the SBD, rock exposure and rock content in different karst landforms, and there is a regular correlation among the different karst landforms: the higher the SBD is, the lower the rock exposure and rock content are.

#### 3.1.2. The Concentration Statistics of SOC in Different Karst Landforms

The concentrations of SOC in the top several soil horizons under different karst landforms are listed in Table 1. Samples decrease with soil depth because the soil thickness in the karst area is thin and discontinuous, resulting in the thickness of some soil layers being less than 50 cm. The SOC content decreased with soil depth, and the mean SOC contents in the 0–5, 5–10, 10–15, 15–20, 20–30, 30–40 and 40–50 cm soil layers were 31.02, 26.86, 23.12, 20.29, 16.32, 11.79 and 9.39 g·kg^−1^, respectively. The coefficient of variation of the SOC in each soil layer is highly variable, which indicates that there are large changes in SOC in each soil layer. According to the skewness and kurtosis information, all the skewness values are positive, which indicates that skewness exhibits a certain degree of right-side bias, and there is SOC enrichment in some local karst regions. Most of the kurtosis data are greater than zero, and the higher the kurtosis value is, the steeper the peak, which reveals that the distribution of SOC may present a cone peak pattern.

Obviously, there is a large discrepancy in the SOC in different soil horizons in karst mountainous areas, and a large discrepancy also exists in the SOC in the same soil layer.

#### 3.1.3. The Spatial Distribution Characteristics of SOC in Different Karst Landforms

**Figure 3:**
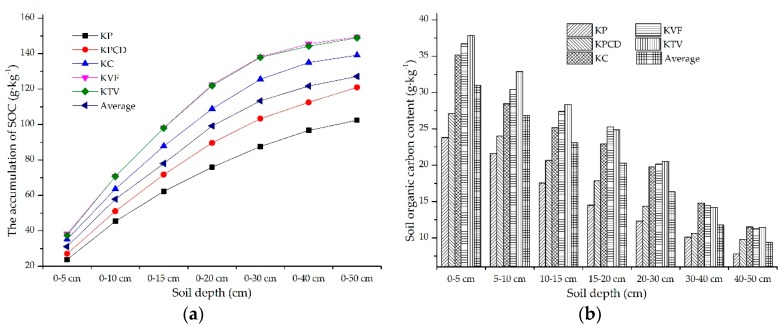
Spatial distribution of soil organic content (SOC) in different landform types with increasing soil depth: (**a**) shows the accumulation of SOC and (**b**) is the content of SOC in a single soil layer.

There were large discrepancies in the SOC among the different karst landform types, and these spatial distribution characteristics of SOC are shown in Figure 3. The accumulation of SOC content in different karst landforms gradually increased with increasing soil depth, and in descending order, the range of the increase in the SOC content was KVF > KTV > KC > KPCD > KP. The average values of the ranges of the increases in SOC in the KVF, KTV and KC were higher than the mean values, and those of both the KPCD and KP were lower than the average values. The range of the increase in SOC in the KVF was highly similar to that in the KTV. In the 0–5 cm soil layer, the range of the SOC contents among the different karst landforms was only 14.50 g·kg^−1^. However, the range of SOC accumulation in the 0–50 cm soil layer reached 46.98 g·kg^−1^. The discrepancy in the SOC accumulation value gradually increased with increasing soil thickness (Figure 3a). The SOC content in a single soil layer gradually decreased with increasing soil depth, and the range of the SOC content in the 0–5 cm soil layer in the different karst landforms was 14.50 g·kg^−1^, but in the 40–50 cm soil layer, the range of the SOC content was only 3.73 g·kg^−1^. The discrepancy of the SOC in a single soil layer in the different karst landforms gradually decreased with increasing soil depth, and this discrepancy of SOC mainly occurred in the upper soil layer. In descending order, the SOC contents in the 0–5, 5–10, 10–15 and 20–30 cm soil layers were KTV > KVF > KC > KPCD > KP, and the SOC contents in the 15–20 cm soil layer in the different karst landforms were KVF > KTV > KC > KPCD > KP in descending order. In descending order, the SOC contents in the 30–40 cm soil layer were KC > KVF > KTV > KPCD > KP, and the order of KC, KPCD and KP was the same as that in the 30–40 cm soil layer, and there was limited discrepancy in the SOC contents in the KTV, KVF and KC (Figure 3b).

### 3.2. The SOC Response to Different Environmental Factors in Different Karst Landforms

#### 3.2.1. The Correlation among SOC and SBD, Rock Exposure and Rock Content

**Figure 4:**
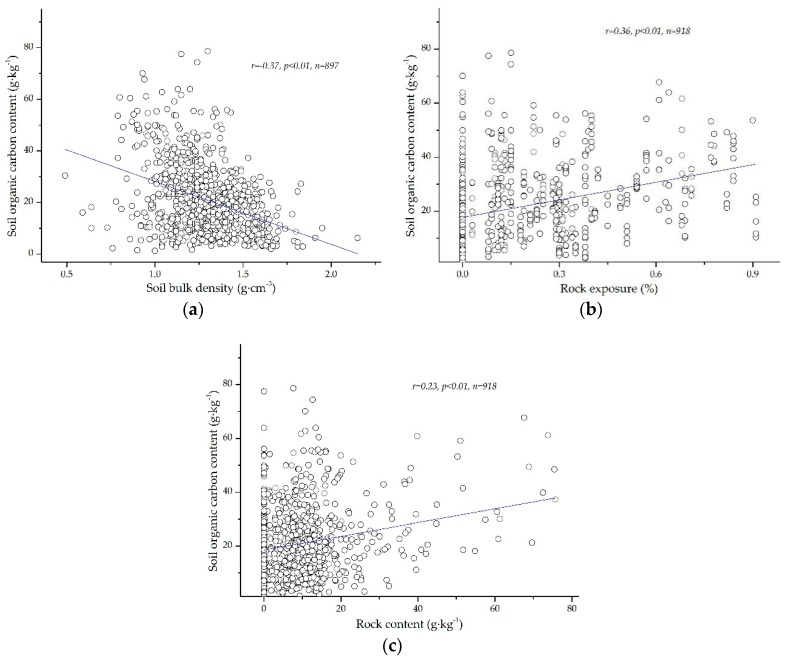
The correlations among soil organic carbon (SOC) content and soil bulk density (SBD), rock exposure and rock content: (**a**), (**b**) and (**c**) represent the correlation among the SOC and SBD, rock exposure and rock content, respectively.

The high spatial heterogeneity of SBD, rock exposure and rock content are important characteristics of soil in different karst landforms, and their coefficient of variation ranges are 0.15–0.26, 0.48–0.80 and 0.63–0.88, respectively. The spatial heterogeneity of the rock content is more than that of the rock exposure rate, and they are both highly variable. The spatial heterogeneity of the SBD is the lowest, and it has moderate variation and low variability. In the topsoil, the relationship between the SOC content and SBD is negatively correlated (*r* = −0.37, *p* < 0.01, *n* = 897); the relationship between the SOC content and rock exposure rate is positively correlated (*r* = 0.36, *p* < 0.01, *n* = 918), and the relationship between the SOC content and rock content is positively correlated (*r* = 0.23, *p* < 0.01, *n* = 918). The correlation information among the SOC content and SBD, rock exposure rate and rock content are listed in Figure 4. In the topsoil, land use is a determining factor of the effects on SOC [27]. SBD, rock exposure and rock content are important factors affecting land, resulting in land being used for different functions. Therefore, SBD, rock exposure and rock content are indirect factors affecting the SOC spatial distribution characteristics.

#### 3.2.2. Effects of Attached Vegetation on SOC in Topsoil

**Figure 5:**
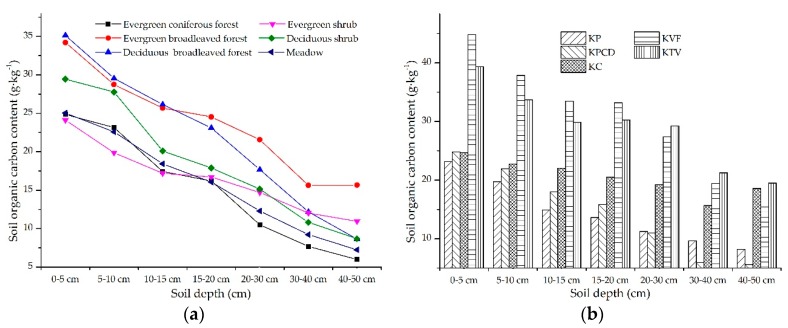

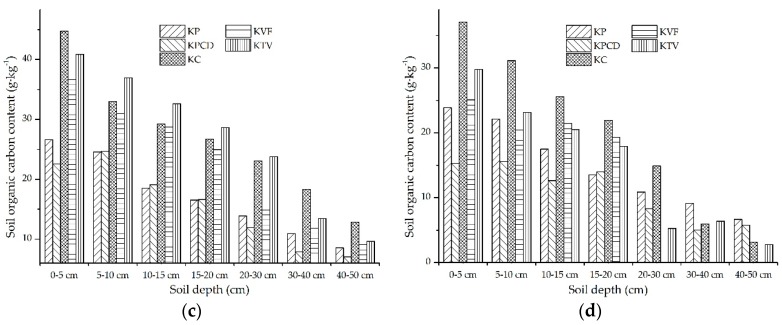
The spatial distribution characteristics of soil organic carbon (SOC) under different vegetation types: (**a**) shows the SOC content in topsoil under different vegetation; (**b**), (**c**) and (**d**) represent the SOC content in topsoil in different karst landforms under evergreen forest, deciduous forest and meadows, respectively.

According to the vegetation at the sampling sites, the plant types were divided into evergreen coniferous forest, evergreen broadleaved forest, deciduous broadleaved forest, evergreen shrub, deciduous shrub and meadows. The spatial distribution characteristics of SOC in topsoil under different vegetation are listed in Figure 5. The SOC contents under soil under different vegetation types gradually decrease with increasing depth in the topsoil, and the SOC contents in the upper soil layer under evergreen broadleaved forest, deciduous broadleaved forest and deciduous shrub are distinctly higher than those under other vegetation types. The SOC exhibits similar distribution characteristics among evergreen coniferous forests, evergreen shrubs and meadows (Figure 5a). To further reveal the spatial heterogeneity of the SOC in the topsoil in different karst landforms under different vegetation types, all vegetation was divided into 3 types: evergreen forest, deciduous forest and meadows. Among them, evergreen coniferous forest, evergreen broadleaved forest and evergreen shrub were included in evergreen forest, and deciduous broadleaved forest and deciduous shrubs were included in deciduous forest. The distribution of SOC in the different karst landforms under evergreen forest showed that the spatial distribution characteristics of SOC in the 0–5, 5–10, 10–15 and 15–20 cm soil layers were similar, and the SOC contents in the different karst landforms in descending order were KVF > KTV > KC > KPCD > KP. The spatial distributions of SOC in the 23–30 and 30–40 cm soil layers were slightly different than those in the 0–5, 5–10, 10–15 and 15–20 cm soil layers, and the SOC contents in the different karst landforms in descending order were KTV > KVF > KC > KP > KPCD. In the 40–50 cm soil layer, the SOC contents in the different karst landforms in descending order were KTV > KC > KVF > KP > KPCD, and the SOC contents in the KP and KPCD were lower than those in the other karst landforms (Figure 5b). The spatial distribution characteristics of SOC in the different karst landforms under deciduous forest showed that in the 0–5 cm soil layer, the SOC content presented gradually decreasing characteristics on three levels. The KC and KTV were in the first level, and the SOC content in the KC was greater than that in the KTV. The KVF was in the second level, the KPCD and KP were in the third level, and the SOC content in the KP was higher than that in the KPCD. The regular SOC contents in the different karst landforms in the 5–10, 10–15 and 15–20 cm soil layers were similar, and the descending order was KTV > KC > KVF > KPCD > KP. In the 20–30 cm soil layer, the SOC content showed regular gradual decreases in two levels. The first level included the KTV and KC, the second level included the KP, KPCD and KVF, and the SOC content in the KVF was slightly higher than that in the KP. In the 40–50 cm soil layer, the descending order of the SOC contents in the different karst landforms was KC > KTV > KVF > KP > KPCD. The KC was in the first level, and the content of SOC in the KC was significantly higher than that in the other karst landforms. The KVF, KP, KPCD and KTV were in the second level, and they had similar SOC contents (Figure 5c).

The spatial heterogeneity of SOC in different karst landforms under meadows showed that similar SOC contents were regularly distributed in the 0–5 and 5–10 cm soil layers, the SOC content was highest in the KC and lowest in the KPCD, and there were small discrepancies in the SOC contents among the KTV, KVF and KP. The same descending order of the SOC content existed in the 10–15 and 15–20 cm soil layers, and the SOC content in the KC was obviously higher than that in the other karst landforms. The SOC content in the KPCD was significantly lower than that in the other karst landforms. The SOC content in the 20–30, 30–40 and 40–50 cm soil layers did not exhibit a regular spatial distribution; however, the data on the soil samples in the KVF were lost. In descending order, the SOC contents in the different karst landforms in the 20–30 cm soil layer were KC > KP > KPCD > KTV, the order in the 30–40 cm soil layer was KP > KTV > KC > KPCD, and the descending order of the SOC contents in the 40–50 cm soil layer in the different karst landforms was KP > KPCD > KC > KPCD.

#### 3.2.3. The SOC in Topsoil in Response to Land Use

**Figure 6:**
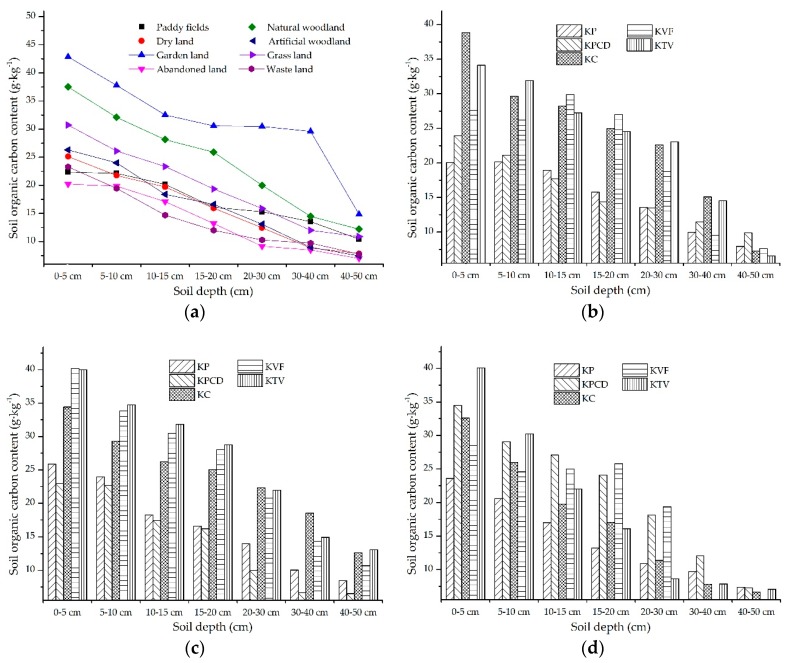
The spatial distribution characteristics of the soil organic carbon (SOC) content under different land uses: (**a**) shows the content of SOC under different land uses; (**b**), (**c**) and (**d**) represent the SOC content in topsoil in different karst landforms in agricultural land, forestry land and unused land, respectively.

According to the soil samples taken from different land uses, the soil from the sampling points was divided into paddy fields, dry land, garden land, abandoned farmland, natural woodland, artificial woodland, grassland and waste land. The discrepancy in the distribution of SOC in topsoil under different land uses is shown in Figure 6a. The SOC content in the topsoil under all different land uses gradually decreased with increasing soil depth, and the SOC contents in garden land and natural woodland were significantly higher than those in the other land uses. There were few discrepancies in the SOC contents among grassland, artificial woodland, dry land, waste land, paddy field and abandoned farmland, and there was no obvious descending order in the soil from the different land uses. To analyze the diversity of the SOC contents in different land uses in the different karst landforms, the land use was divided into three main types of agricultural land, forestry land and unused land. Among them, paddy fields, dry land and garden land were included in agricultural land; natural woodland and artificial woodland were included in forestry land; and abandoned farmland, grassland and waste land were included in unused land. The discrepancies in the SOC contents among the different karst landforms were compared in the three land use types, and the spatial distribution characteristics of SOC in agricultural land, forestry land and unused land are individually shown in Figure 6b–d. The diverse distribution of SOC content in the topsoil in agricultural land is shown in Figure 6b. In the 0–5 cm soil layer, the descending order of SOC contents in the different karst landforms was KC > KTV > KVF > KPCD > KP, and the SOC contents in all the karst landforms could be divided into two levels of gradual decreases, among which the KC and KTV were in the first level, and the second level included the KVF, KPCD and KP. The spatial distribution characteristics of the SOC content in the 5–10 cm soil layer were similar to those in the 20–30 cm soil layer, and the SOC contents in the different karst landforms could be divided into three levels of gradual decreases. Among them, the KTV and KC were in the first level, and the SOC content in the KTV was higher than that in the KC. The KVF was in the second level. The third level included the KP and KPCD, and the SOC contents of both karst landforms were similar. The distributions of SOC contents were the same in the 10–15 and 15–20 cm soil layers. The descending order of the SOC contents in the different karst landforms was KVF > KC > KTV > KP > KPCD, among which the KVF, KTV and KC were in the first level, the KP and KPCD were in the second level, and the SOC content in the first level was significantly higher than that in the second level. The descending order of the SOC contents in the different karst landforms in the 30–40 cm soil layer was KC > KTV > KPCD > KP > KVF, and the SOC contents in the KC and KTV were obviously higher than those in the other karst landforms. There was little discrepancy in the SOC contents among the different karst landforms in the 40–50 cm soil layer, and the descending order of SOC contents in the different karst landforms was KPCD > KP > KVF > KC > KTV.

The distribution characteristics of the SOC content in forestry land are shown in Figure 6c. The same regular distribution of the SOC content existed in the 0–5, 5–10, 10–15 and 15–20 cm soil layers, and the SOC contents in the different karst landforms gradually decreased from KTV > KVF > KC > KP > KPCD. The SOC content in the different karst landforms could be divided into two levels with gradual decreases: the first level included the KTV, KVF and KC, the second level included the KP and KPCD, and the SOC content in the first level was obviously higher than that in the second level. The spatial distributions of the SOC contents in the 20–30, 30–40 and 40–50 cm soil layers were similar in the different karst landforms, and the descending order of SOC contents in the different karst landforms was divided into three levels. The first level included the KC, KTV and KVF; the KP and KPCD were in the second level and the third level, respectively. The SOC content in the first level was significantly higher than that in the second and third levels. The distribution characteristics of SOC contents in unused land are shown in Figure 6d. In the 0–5 and 5–10 cm soil layers, the descending order of SOC contents in the different karst landforms was KTV > KPCD > KC > KVF > KP. In the 10–15, 15–20 and 20–30 cm soil layers, a similar regular distribution of the SOC contents existed in the different karst landforms. The SOC contents in the KPCD and KVF were obviously higher than those in the other landforms, and there was limited discrepancy in the SOC contents in the KP, KC and KTV. In the 30–40 and 40–50 cm soil layers, the soil samples were taken from the KP, KPCD, KC and KTV. The descending order of the SOC contents in the 30–40 cm soil layers of the different karst landforms was KPCD > KP > KC ≈ KTV, and the contents of SOC in the karst landforms in the 40–50 cm soil layers were highly similar.

#### 3.2.4. The Response of the SOC Content in Topsoil to Soil Types

**Figure 7:**
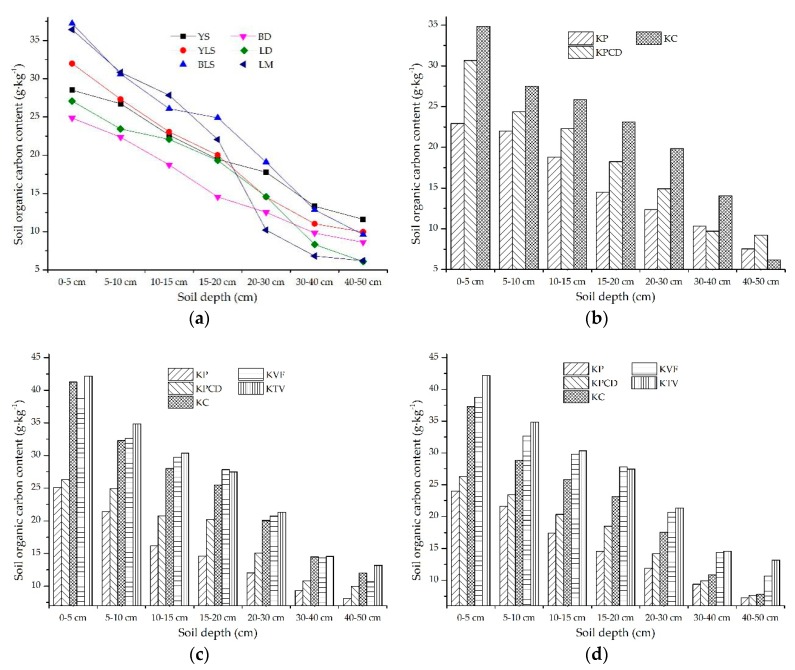
The spatial distribution characteristics of the soil organic carbon (SOC) content in different soil types: (**a**) shows the topsoil SOC content in different soil types; (**b**), (**c**) and (**d**) are the SOC contents in paddy soil, limestone soil and yellow soil, respectively.

The soils were divided into six types: Xanthi-udic Ferralsols (YS), Black Lithomorphic Isohumisols (BLS), Cab Udi Orthic Entisols (YLS), Cab High fertility Orthic Anthrosols (BD), Cab Low fertility Orthic Anthrosols (LD) and large mud field loam (LM) [24]. The spatial heterogeneity of the SOC content in the different soil types is shown in Figure 7a. The SOC content in the 0–5 cm soil layer reached 12.36 g·kg^−1^, but in the 40–50 cm soil layer, the maximum was 5.53 g·kg^−1^. The regular distribution of the SOC contents in the different soil horizons gradually decreased with the increase in soil horizons. In the upper 0–20 cm soil layer, the SOC contents in BLS and LM were obviously higher than those in the other soils, but the SOC content in LM decreased rapidly at a soil depth of 20 cm. There was no obvious regular distribution of SOC in YS, YLS, BD and LD, but there was little discrepancy in the SOC contents in these four soils. To further analyze the spatial discrepancy characteristics of SOC in different soil types in different karst landforms, the six soil types were classified into three soil types: paddy soil, limestone soil and yellow soil. Among them, YLS, BLS, BD and LD were included in limestone soil. LM was considered paddy soil, and YS was considered yellow soil. The spatial discrepancy of the SOC content in the different karst landforms among the three soil types was analyzed, and their distribution characteristics are shown in Figure 7b–d. The paddy soil samples were taken from the KP, KPCD and KC, the regular distribution of SOC was the same in the 0–5, 5–10, 10–15, 15–20 and 20–30 cm soil layers, and the descending order of the SOC contents in the karst landforms was KC > KPCD > KP. In the 30–40 cm soil layer, the SOC content in the KC was obviously higher than that in the KPCD and KP, and the SOC contents in the KP and KPCD were similar. The SOC contents in the KPCD, KP and KC gradually decreased in the 40–50 cm soil layer, among which the decrease in the KPCD was the largest and that in the KC was the smallest (Figure 7b).

The spatial heterogeneity of SOC in limestone soil is shown in Figure 7c. In the 0–5 cm soil layer, the SOC contents in different karst landforms were divided into two gradually descending levels. The first level included the KTV, KC and KVF, the second level included the KPCD and KP, and the SOC content in the second level was significantly lower than that in the first level. The SOC contents exhibited similar patterns in the 5–10, 10–15 and 20–30 cm soil layers, and the descending order of the SOC contents in the different karst landforms was KTV > KVF > KC > KPCD > KP. The descending order of SOC contents in the karst landforms in the 15–20 cm soil layer was similar to that in the 10–15 cm soil layer, but the order of the KVF and KTV changes, and the SOC content in the KVF was slightly higher than that in the KTV. In the 30–40 cm soil layer, the SOC contents in different karst landforms could be divided into two gradually decreasing levels: the first level included the KTV, KVF and KC, the second level included the KP and KPCD, and the SOC content in the first level was obviously higher than that in the second level. In the 40–50 cm soil layer, the descending order of the SOC contents in the different karst landforms was KTV > KC > KVF > KPCD > KP, and there was little discrepancy in the SOC contents in all the karst landforms. The spatial distribution of SOC in yellow soil in different karst landforms is shown in Figure 7d. The regular distribution of the SOC contents in the different karst landforms was similar in all soil layers, and the descending order of the SOC contents in the different karst landforms was KTV > KVF > KC > KPCD > KP. The SOC contents in the different karst landforms in the 0–5 cm soil layer could be divided into two levels, and the SOC content in the first level was significantly higher than that in the second level. The SOC contents in the different karst landforms in the 5–10, 10–15, 15–20 and 20–30 cm soil layers exhibited gradual decreases. In the 30–40 and 40–50 cm soil layers, there was little discrepancy in the SOC contents in the KP, KPCD and KC.

### 3.3. The Analysis of Environmental Factors

#### 3.3.1. Dimension-Reduction Analysis of Impact Factors

The correlation among different environmental factors was analyzed before PCA factor analysis, and the Kaiser–Meyer–Olkin (KMO) measure of sampling adequacy and Bartlett’s test were calculated via SPSS software. The KMO vale ranges between zero and one, and the higher the KMO and the lower the *p*-value of the Bartlett test are, the more significant the correlation is among different factors. The results showed that the KMO value was 0.723, and the *p*-value of Bartlett’s test was much lower than 0.05 (*p* < 0.000), which indicates that the correlation among different environmental factors was significant. The information is shown in Table 2.

The correlation coefficients among the different factors were calculated via Pearson’s method. The correlation was significant at the 0.01 level, such as the correlation among the microenvironment, slope position, slope aspect, slope gradient, altitude, atmospheric pressure, land use, lithology and SBD. The correlation among rock exposure, rock content and SBD was significantly correlated at the 0.01 level as well. In addition, the altitude and atmospheric pressure and the slope aspect and SBD were significantly negatively correlated at the 0.05 level. These correlations indicated that there was a synergistic effect or antagonistic effect among different factors. There were not only linear correlations among the different factors but also more complex nonlinear correlations. To more accurately analyze the response model of factors that have effects on SOC in topsoil, the dimensions of the impact factors were reduced by using PCA in SPSS. The results of the factor analysis of PCA are shown in Table 3. The first four principal components represented 87.018% of the correlation among different factors. The characteristic value of the first principal component was 4.656, and the contribution rate of the first principal component reached 38.804%. The microenvironment, slope position, altitude, land use and lithology had high positive loads, which indicated that the microenvironment, slope position, altitude, land use and lithology played a major role in the first principal axis. The characteristic value and contribution rate of the second principal component were 2.991 and 24.992%, respectively, and the accumulated contribution rate reached 59.88%. The slope aspect had a high positive load, and it played a key role in the second principal axis. The characteristic value and the contribution rate of the third principal component were 1.533 and 12.777%, respectively, and the accumulated contribution rate was 76.502%. The soil type and slope gradient had a high positive load, and the soil type and slope gradient played important roles in the third principal axis. In addition, the characteristic value and contribution rate of the fourth principal component were 1.262 and 10.516%, respectively, and the accumulated contribution was 87.018%. The rock exposure had a high positive load, and it was a key factor in the fourth principal component.

According to the analysis, the dimensionality-reduction model used for the different environmental factors mainly used four alliance models of microenvironment-slope position-altitude-land use-lithology, slope aspect, soil genus-slope gradient and rock exposure. All the different factors affecting the topsoil SOC could be divided into internal factors and external factors. Among these factors, microenvironmental factors, slope position, slope aspect, slope gradient, altitude, atmospheric pressure, land use and rock exposure were categorized as external factors, and soil type, lithology, SBD and rock content were categorized as internal factors. Obviously, the SOC content in topsoil was influenced by the synergistic effects of internal factors and external factors.

#### 3.3.2. The Sorting of Environmental Factors Affecting SOC

**Figure 8:**
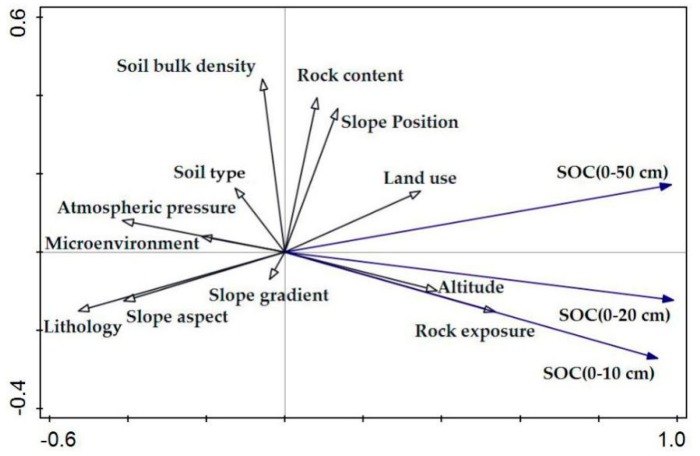
The sorting of correlation between topsoil soil organic carbon (SOC) and different factors.

The influence of environmental factors on the SOC content in topsoil was determined by multivariate analysis. The surface soil was more disturbed than the lower soil layer by external factors, and the surface soil microenvironment was more complex in the surface soil. External factors could be divided into natural factors and human factors. Natural factors included the topsoil microenvironment, climate and vegetation, and human factors included agricultural activities. Therefore, the SOC content in topsoil was influenced by the synergistic effects of different environmental factors. To analyze the different environmental responses to topsoil SOC, the environmental factors were reordered by RDA, and the information is shown in Figure 8. Obviously, the angles among SOC (0–10 cm), SOC (0–20 cm) and SOC (0–50 cm) were lower than 90°, which indicated a positive correlation among the SOC contents in each soil layer in the topsoil. Especially in the 0–10 and 0–20 cm soil layers, the arrow aspects of SOC (0–10 cm) and SOC (0–20 cm) were basically the same, revealing that the topsoil SOC was impacted by similar environmental factors. The SOC content in the topsoil layer was mainly affected by rock exposure, altitude, land use, atmospheric pressure, microenvironment, lithology and slope aspects. Rock exposure, altitude and land use had positive effects on the topsoil SOC, and the microenvironment, atmospheric pressure, lithology and slope aspect had negative effects on the topsoil SOC. In addition, in descending order, the positive effect factors on the SOC contents in the 0–10 and 0–20 cm soil layers were rock exposure > altitude > land use, and that in the 0–50 cm soil layer was rock exposure > altitude > land use. In descending order, the negative impact factors were lithology > atmospheric pressure > slope aspect > microenvironment. In summary, the topsoil SOC content was mainly positively affected by external factors and human factors, and it was mainly negatively affected by the synergy of internal factors and natural factors. Overall, the positive promotion or negative restraint occurred via the synergistic effect of different factors, and the external factors had a main impact on the SOC content in the topsoil layer.

## 4. Discussion

### 4.1. The Difference in Topsoil SOC Contents in Different Karst Landforms and Its Influence Mechanism

The SOC is not permanently fixed in soils because of the long-term dynamic balance of outputs and inputs [32]. The spatial distribution of SOC was affected by many factors, and the key factors differed in different regions [33,34]. According to the information in Table 1, the SOC content gradually decreases with increasing soil depth, which indicates that the SOC content in the upper soil layer is higher than that in the lower soil layer. There is high heterogeneity in the spatial distribution of SOC in different karst landforms, and the SOC content in each soil layer of the KTV, KVF and KC is obviously higher than that in the KP and KPCD. The reasons for the SOC contents in the KTV, KVF and KC being higher than those in the KP and KPCD are as follows. First, the SOC contents in topsoil with different vegetation, land use and soil types in the KVF, KTV and KC are generally higher than those in the KPCD and KP because most soil samples from the KVF, KTV and KC were collected from land under different forest types, and the amount of soil humus in the topsoil layer in forestland is higher than that in other land uses. Second, the KVF is a protected area that is less disturbed by humans, so the microenvironment on the surface in the KVF is more stable than that in the other karst landforms. In addition, the land in the KTV and KC has abundant vegetation, and agricultural land is an important land use type. The land in the KTV and KC is strongly influenced by human beings, chemical fertilizer is widely applied on agricultural soil, and the concentrations of calcium ions and nitrogen ions in the soil are high. Calcium (Ca^2+^) and nitrogen ions in chemical fertilizer can control the SOC content, and the higher the contents of Ca^2+^ and N are, the higher the SOC content in the soil [35]. The more organic matter that is present in the soil under forest, the higher the SOC content [10], which results in the SOC content in the KTV, KVF and KC being generally higher than that in the other karst landforms. Moreover, grassland is the main land use type in the KPCD and KP, the ecosystem structure is simple, and the organic matter is low in the surface soil, which results in the SOC contents in the KP and KPCD being lower than those in the other karst landforms. Third, the relationship between SBD and the SOC content in topsoil is negative, but the relationships between the SOC content and rock exposure and SOC content and rock content are positive. According to the information in Figure 2, the soil bulk densities in the KPCD and KP are higher than those in the other karst landforms, but the rock exposure and rock content in the soil of the KVF, KTV and KC are generally higher than those in the soil of the KPCD and KP. Therefore, the contents of SOC in the KVF, KTV and KC are higher than those in the other karst landforms. These results agree with the results of RDA that indicated that rock exposure is an important factor with a positive effect on SOC. These results are similar to the results of Yan [9] and Huang [36], in which the SBD was found to have a negative effect on SOC and the relationship between rock exposure and SOC content was found to have a positive correlation. Finally, the external factors are complex, and the internal factors are stable relative to the environment of the soil surface. Therefore, in different vegetation, land use and soil types, there is a high discrepancy in the SOC contents in the upper soil in different karst landforms, but there is a small discrepancy in the SOC contents in the low soil layers. In summary, the SOC contents in the KVF, KTV and KC were generally higher than those in the KC and KPCD, the SOC at the surface was impacted by the synergistic effect of multiple factors, and the SOC in the lower soil was influenced by internal factors. In addition, rock exposure and land use are key factors that positively promote SOC, and SBD is the main negative factor affecting SOC.

### 4.2. Comparison with Other Karst Mountainous Areas

Currently, the research on SOC during the karst rocky desertification processes in Guizhou Province has mainly focused on karst basins. For example, the assessment by Huang et al. indicated that the SOC content and SOC density in the 0–20 cm soil layer in karst basins reached 25.07 g·kg^−1^ and 4.27 kg·m^−2^, respectively [36]. Zhang et al. estimated that the total SOC storage in the 0–10 cm and 0–100 cm soil horizons in the Houzhai River Basin were approximately 2.65 × 10^8^ and 5.39 × 10^8^ kg C, respectively. In addition, Zhang noted that the SOC content significantly decreased at a depth of 0–50 cm, but the SOC content slowly decreased in the 50–100 cm soil layer, indicating that a depth of 50 cm is a turning point of the SOC content [10]. Zhang et al. investigated the patterns of the spatiotemporal variability in SOC storage in a karst basin, and the results showed that the SOC content was 21.98 g/kg in 1980 and 25.07 g·kg^−1^ in 2015, with an increase of 3.09 g·kg^−1^ (14.58%) [11]. Zhang noted that the key factors that could affect the spatial distribution of SOC in karst basins include soil type, land use and major environmental factors. 

The SOC contents in topsoil exhibits high discrepancy in different karst landforms, and the coefficient of variation of the SOC content in each soil layer in all the different karst landforms is highly variable. These results are similar to those of Zhang in that the SOC content in the 0–50 cm soil layer is easily disturbed by human activities or other external environmental factors, so large changes in SOC occur in the topsoil layer, but this difference is gradually reduced with increasing soil depth [11]. This research was compared with research in the karst catchment in Puding, Guizhou Province. The SOC contents in the 0–20 cm soil layer in the KTV, KVF and KC are obviously higher than those found by Zhang, who found that the SOC content in the karst catchment was 25.07 g·kg^−1^, and the SOC contents in the KPCD and KP were slightly lower than those in the catchment [10]. Zhang noted that the SOC in the catchment is mainly disturbed by soil type, rock exposure, land use and environmental factors, and these results are similar to the results of this study, which found that the SOC in the topsoil layer in different karst landforms is impacted by external factors such as land use, rock exposure and altitude [10,15]. The results of this study were compared with Zhang’s results, which showed that the SOC content range is similar among different karst landforms, but the main impact factors may be different. This study was compared with the different soil types in small karst watersheds, and the SOC contents in different soil types are similar in that the SOC contents in Black Lithomorphic Isohumisols and Cab Udi Orthic Entisols are significantly higher than those in other soil types. The SOC contents in different karst landforms in this study are slightly lower than those in the small karst watersheds because the SOC contents in different soil types in small karst watersheds are generally higher than those in this research [24]. The SOC contents in different karst landforms can be compared with those in karst basins. The regular spatial distribution of the SOC in different soil horizons in different karst landforms is similar to that in the Houzhai River Basin, and the spatial heterogeneity of SOC in the upper soil layers is higher than that in the lower soil layers [27]. The SOC content gradually decreases with increasing soil depth under different land use types and vegetation types. The results of this study are comparable to the results of Huang’s research. Only the SOC content in the KVF is slightly higher than that in the karst basin, and the SOC contents in the other karst landforms are lower than those in the karst basin to different degrees. This difference occurs because most soil samples from Huang’s research were mainly taken from land under different vegetation types, such as arbor forestland, shrub lands and arbor-shrub mixed forestland, and these forestlands are abundant in organic matter, which results in SOC-rich soil [25,27]. Moreover, the sorting of land use and vegetation in this study is less detailed than that in Huang’s research. The main purpose of this study was to clearly compare the discrepancy in the SOC contents in different karst landforms, while the main aim of Huang’s study was to analyze the spatial distribution characteristics of SOC under different land use and vegetation conditions [25,27]. In summary, the SOC content in soil under forests is generally greater than that under other land uses [37], and the SOC content in limestone soil is higher than that in other soil types [38]. Therefore, the SOC contents in different vegetation, land use and soil types in the KVF, KTV and KC are generally higher than those in other karst landforms.

## 5. Conclusions

The SOC contents in the 0–50 cm topsoil layer in different karst landforms are between 7.76 and 38.29 g·kg^−^^1^, and the SOC exhibits similar regular distribution among different karst landforms in which the SOC content gradually decreases with increasing soil depth. There is a large discrepancy in the coefficient of variation of the SOC in topsoil; the change in the SOC content in each soil layer among different karst landforms is highly variable, while the difference in the SOC content in the upper soil layer is higher than that in the lower soil layer. In descending order, the SOC contents in the different karst landforms are KTV > KVF > KC > KPCD > KP, and the SOC contents in the KTV and KVF are obviously higher than those in the other karst landforms. SOC is impacted by the synergy or restraint of different environmental factors, among which land use, rock exposure and altitude positively promote SOC in soil, and SBD is the main factor that has a negative effect on SOC. The SOC contents in forestland and agricultural land are generally higher than those of other land use types, and the higher the calcium ion or organic matter content is, the higher the SOC content. The surface SOC is mainly influenced by external factors, and the microenvironment of the topsoil layer is more easily disturbed by human activities, which results in the reconstruction of the spatial distribution of SOC in different karst landforms being influenced by human activities. The soil layer thickness in karst mountainous areas is generally thinner than that in non-karst areas and in fragile ecosystems in different karst areas. Karst areas are not suitable for large-scale agricultural production. Therefore, the method of reducing human disturbance and afforestation to increase organic matter is a feasible way to increase the SOC contents in karst areas. Because of the high spatial heterogeneity of the ecological environments in different karst areas, the SOC content is impacted by multiple factors, and the key factors are also different in different study areas. Therefore, obtaining a more accurate method to assess the SOC contents in different karst landform regions requires further research.

## Figures and Tables

**Table 1:** Descriptive statistics of the soil organic carbon (SOC) content in soil horizons.

Soil Horizons	Samples (n)	Maximum	Minimum	Mean	CV (%)	Skewness	Kurtosis
		g·kg^−1^					
0–5 cm	152	94.11	9.52	31.02	0.52	1.11	1.14
5–10 cm	152	78.58	9.59	26.86	0.46	1.01	1.25
10–15 cm	146	54.82	5.81	23.12	0.48	0.72	−0.08
15–20 cm	143	54.86	3.14	20.29	0.56	0.90	0.40
20–30 cm	132	55.82	2.59	16.32	0.63	1.47	2.60
30–40 cm	108	48.55	1.64	11.79	0.68	1.81	4.38
40–50 cm	87	36.34	1.22	9.39	0.69	1.95	4.88

Note: CV is the coefficient of variation.

**Table 2:** Kaiser–Meyer–Olkin (KMO) and Bartlett’s test.

Kaiser–Meyer–Olkin Measure of Sampling Adequacy	KMO Coefficient	0.723
Bartlett’s test of sphericity	Approximately chi-square	168.792
	df	136
	Sig.	0.000

**Table 3:** The principal component analysis (PCA) of environmental factors via the maximum variance method.

Environmental Factors	Component I	Component II	Component III	Component IV
Soil genus	−0.371	−0.370	0.742	−0.367
Microenvironment	0.686	0.518	0.190	0.214
Slope position	0.807	−0.503	0.017	−0.133
Slope aspect	0.378	0.891	0.044	−0.150
Slope gradient	0.506	0.556	0.607	0.115
Altitude	0.858	−0.369	−0.259	0.108
Atmospheric pressure	−0.840	0.371	0.314	−0.129
Land use	0.605	−0.361	0.290	0.480
Lithology	0.686	0.501	−0.301	−0.385
Rock exposure	−0.532	0.175	−0.016	0.781
Soil bulk density	−0.427	−0.444	−0.236	−0.089
Rock content	0.505	−0.590	0.425	−0.057
Characteristic value	4.656	2.991	1.533	1.262
Contribution rate of variance (%)	38.804	24.922	12.777	10.516
Accumulated contribution rate (%)	38.804	63.762	76.502	87.018
